# Tumor Infiltrating CD8^+^ and Foxp3^+^ Lymphocytes Correlate to Clinical Outcome and Human Papillomavirus (HPV) Status in Tonsillar Cancer

**DOI:** 10.1371/journal.pone.0038711

**Published:** 2012-06-12

**Authors:** Anders Näsman, Mircea Romanitan, Cecilia Nordfors, Nathalie Grün, Hemming Johansson, Lalle Hammarstedt, Linda Marklund, Eva Munck-Wikland, Tina Dalianis, Torbjörn Ramqvist

**Affiliations:** 1 Department of Oncology-Pathology, Karolinska Institutet, Stockholm, Sweden; 2 Department of Oto-Rhino-Laryngology, Head and Neck Surgery, Karolinska Institutet, Karolinska University Hospital, Stockholm, Sweden; John Hopkins Medical School, United States of America

## Abstract

**Background:**

Human papillomavirus (HPV) is a causative factor for tonsillar squamous cell carcinoma (TSCC) and patients with HPV positive (HPV^+^) TSCC have a better clinical outcome than those with HPV negative (HPV^−^) TSCC. However, since not all patients with HPV^+^TSCC respond to treatment, additional biomarkers are needed together with HPV status to better predict response to therapy and to individualize treatment. For this purpose, we examined whether the number of tumor infiltrating cytotoxic and regulatory T-cells in TSCC correlated to HPV status and to clinical outcome.

**Methods:**

Formalin fixed paraffin embedded TSCC, previously analysed for HPV DNA, derived from 83 patients, were divided into four groups depending on the HPV status of the tumor and clinical outcome. Tumors were stained by immunohistochemistry and evaluated for the number of infiltrating cytotoxic (CD8^+^) and regulatory (Foxp3^+^) T-cells.

**Results:**

A high CD8^+^ T-cell infiltration was significantly positively correlated to a good clinical outcome in both patients with HPV^+^ and HPV^-^ TSCC patients. Similarly, a high CD8^+^/Foxp3^+^ TIL ratio was correlated to a 3-year disease free survival. Furthermore, HPV^+^TSCC had in comparison to HPV^−^TSCC, higher numbers of infiltrating CD8^+^ and Foxp3^+^ T-cells.

**Conclusions:**

In conclusion, a positive correlation between a high number of infiltrating CD8^+^ cells and clinical outcome indicates that CD8^+^ cells may contribute to a beneficial clinical outcome in TSCC patients, and may potentially serve as a biomarker. Likewise, the CD8^+^/Foxp3^+^cell ratio can potentially be used for the same purpose.

## Introduction

Recent studies from Europe and the US have reported an increase in the incidence of oropharyngeal squamous cell carcinoma (OSCC), particularly for tonsillar squamous cell carcinoma (TSCC). This increase has been attributed to an increased prevalence of human papillomavirus (HPV) infection [1], [2], [3], [4]. Accordingly, a 7-fold increase in HPV-positive (HPV^+^) TSCC, in parallel to a decline in HPV-negative (HPV^−^) TSCC has been demonstrated for patients in Stockholm, Sweden, the last three decades [3] and a similar trend has been demonstrated in the US [2]. Importantly also, HPV has been shown to be a favorable prognostic factor for patients with OSCC including TSCC [5], [6], [7], [8]. However, not all patients with HPV^+^TSCC fare well and thus, although HPV by itself is an important prognostic marker, additional biomarkers are needed in TSCC to better predict treatment response before individualized treatment can be implemented in clinical practice.

It has been suggested that HPV^+^ and HPV^−^TSCC are two different tumor disease entities with different tumor and patient characteristics. Whereas HPV associated tumors, including HPV^+^TSCC are driven by viral oncogenes, e.g. E6 and E7, and have fewer cellular mutations, HPV^−^TSCC develop by accumulation of genetic changes mainly caused by environmental factors such as smoking [9]. As foreign antigens, E6 and E7 may act as potential targets for an immune response against HPV induced tumors and *in vitro* studies have shown that T-cells isolated from HPV^+^ cervical cancer can recognize and kill E6 and E7 expressing tumor cells [10]. Moreover, tumor specific T-cells are detected in most cervical cancer patients, although at low and insufficient levels [11], [12] and the presence of tumor infiltrating lymphocytes (TILs) has been linked to a better prognosis [13], [14]. This has also been observed for other tumor types with no known viral association [15], [16]. However, TILs may act as a double-edged sword. While a pronounced infiltration of CD8^+^ lymphocytes has been linked to a favorable prognosis in many malignancies, the role of infiltrating T regulatory cells (Tregs) (often defined as Foxp3^+^ lymphocytes) is often the reverse [17]. Tregs may promote tumor progression, and increased levels of Tregs have been observed in patients with a variety of tumors and linked to a worse prognosis [18], [19], although there are exceptions where Treg infiltration has been linked to a favorable prognosis [20], [21]. Nevertheless, the role of infiltrating CD8^+^ and Foxp3^+^ lymphocytes has, to our knowledge, not been investigated in TSCC in correlation to patient outcome and HPV status.

**Table 1:** Characteristics of patients and tumors included in the study.

		**Group A**		**Group B**		**Group C**		**Group D**	
		**HPV^+^ good** 1		**HPV^+^ poor** 1		**HPV^−^ good** 1		**HPV^−^ poor** 1	
**Total**		**31**		**21**		**11**		**20**	
		n	%	n	%	n	%	n	%
**Sex**									
	*male*	21	68%	15	71%	9	82%	17	85%
	*female*	10	32%	6	29%	2	18%	3	15%
**Age** (years)									
	*mean*	56.1		62.3		60.9		64.4	
	*median*	56		62		59		61.5	
**Stage**									
	*I*	0	0%	0	0%	4	36%	0	0%
	*II*	2	6%	1	5%	2	18%	1	5%
	*III*	13	42%	8	38%	2	18%	5	25%
	*IV*	16	52%	12	57%	3	27%	14	70%
**Differentiation**									
	*low*	20	65%	14	67%	6	55%	9	45%
	*medium*	11	35%	6	29%	5	45%	8	40%
	*high*	0	0%	1	5%	0	0%	3	15%
**Treatment**									
	*RT* 2	31	100%	20	95%	11	100%	20	100%
	*CRT* 2	0	0%	1	5%	0	0%	0	0%

1 Good and poor denote clinical outcome.

2 Abbreviations: RT, radiotherapy; CRT, chemoradiotherapy.

**Table 2:** Study sample and all patients with HPV^+^TSCC and a good clinical outcome, diagnosed in Stockholm between 2000–2006.

		Sample		All patients	
		n=31		n=109	
		n	%	n	%
**Sex**					
	*male*	21	68%	84	77%
	*female*	10	32%	25	23%
**Stage**					
	*I*	0	0%	1	1%
	*II*	2	6%	9	8%
	*III*	13	42%	36	33%
	*IV*	16	52%	63	58%
**Differentiation**					
	*low*	20	65%	71	65%
	*medium*	11	35%	37	34%
	*high*	0	0%	1	1%
**Treatment**					
	*RT* 1	31	100%	106	97%
	*CRT* 1	0	0%	3	3%
**Age** (years)					
	*mean*	56.1		58.2	
	*median*	56		57	

1 Abbreviations: RT, radiotherapy; CRT, chemoradiotherapy.

The purpose of this study was to examine the presence of tumor infiltrating CD8^+^ and Foxp3^+^ T-cells in TSCC in relation to clinical outcome and tumor HPV status, and to establish if these TILs can potentially be used in the clinic as biomarkers either alone, or together with the HPV status of the tumor to predict clinical outcome.

**Figure 1:**
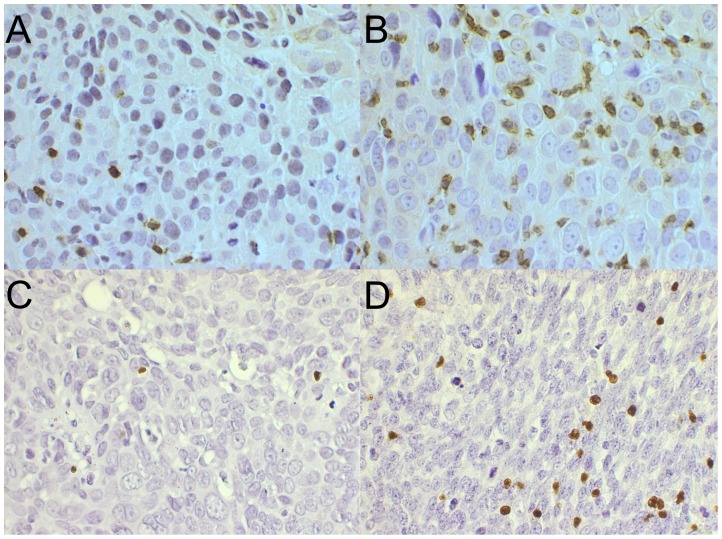
Examples of TSCC stained for tumor infiltrating CD8^+^ and Foxp3^+^ cells. TSCC with (A) low and (B) high CD8^+^ infiltration and (C) low and (D) high Foxp3^+^ infiltration.

**Table 3:** Number of Foxp3^+^ and CD8^+^ T-cells and CD8^+^/Foxp3^+^ cell ratio by clinical outcome and tumor HPV status.

		Clinical outcome		Unadjusted effects^b^		Effects^b^ adjusted for HPV status	
Factor	HPV status^a^	Good (n=42)	Poor (n=41)	Mean difference (95% CI)	P-value^c^	Mean difference (95% CI)	P-value^c^
**Mean CD8^+^ TIL value**	Positive	61.4 (31)	21.0 (21)	40.4 (15.8 to 65.0)	0.002		
	Negative	19.4 (11)	4.7 (20)	14.7 (6.2 to 23.1)	0.001		
					0.12^d^		
	Total	50.4 (42)	12.8 (41)	37.6 (21.3 to 53.8)	<0.001	30.9 (15.1 to 46.7)	<0.001
**Mean Foxp3^+^ TIL value**	Positive	33.7 (31)	32.5 (21)	1.2 (−16.0 to 18.4)	0.89		
	Negative	11.6 (11)	16.4 (20)	−4.7 (−12.2 to 21.6)	0.57		
					0.65^d^		
	Total	27.9 (42)	24.6 (41)	3.3 (−9.2 to 15.8)	0.60	−0.95 (−13.3 to 11.4)	0.88
**Mean CD8^+^/Foxp3^+^ ratio**	Positive	3.0 (31)	1.6 (21)	1.4 (−0.4 to 3.1)	0.13		
	Negative	8.2 (11)	1.1 (20)	7.0 (−1.5 to 15.6)	0.10		
					0.10^d^		
	Total	4.3 (42)	1.4 (41)	3.0 (−0.3 to 6.2)	0.07	3.5 (0.15 to 6.8)	0.04

a HPV data obtained from previous studies (3, 6, 25).

b Effects of HPV-status (negative versus positive) estimated using linear regression.

c P-values refers to F-tests.

d Overall test for effect modification (i.e. interaction between HPV status and clinical outcome).

## Materials and Methods

### Ethics Statement

The study was approved by the Ethical Committee at Karolinska Institutet, Stockholm, Sweden, according to the ethical permissions 2005/431-31/4, 2005/1330-32 and 2009/1278-31/4. Written informed consent was obtained from all participants in the study.

**Figure 2:**
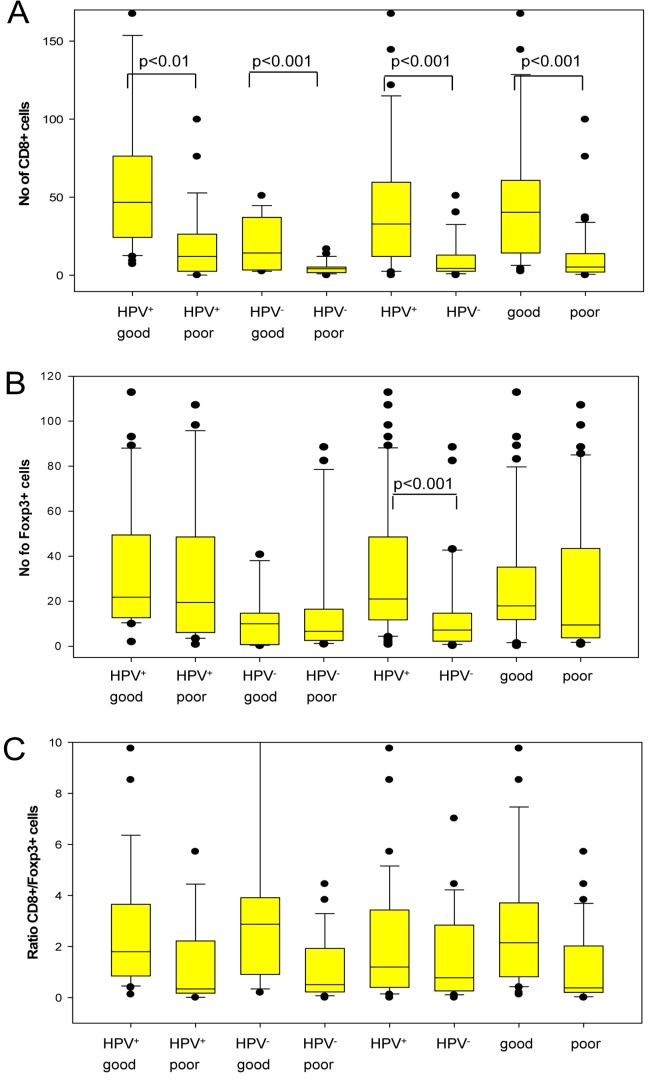
Box plots presenting tumor infiltrating CD8^+^ and Foxp3^+^ cells for different groups of TSCC. A) number of CD8^+^ TILs, B) number Foxp3^+^ TILs and C) the CD8^+^/Foxp3^+^ cell ratio. “Good” and “poor” denotes clinical outcome. In addition to the four TSCC groups defined in the Methods section, different combinations of groups have also been compared. Thus, “HPV^+^ good” corresponds to group A; “HPV^+^ poor” to group B; “HPV^−^ good” to group C; “HPV^−^ poor” to group D; HPV^+^ to groups A+B, HPV^−^ to groups C+D; “good” to groups A+C; and “poor” to groups B+D. In order to better visualize the details in the lower part of Figure 1C the upper part of the diagram including the upper whisker (at a ratio of 80) was omitted.

**Figure 3:**
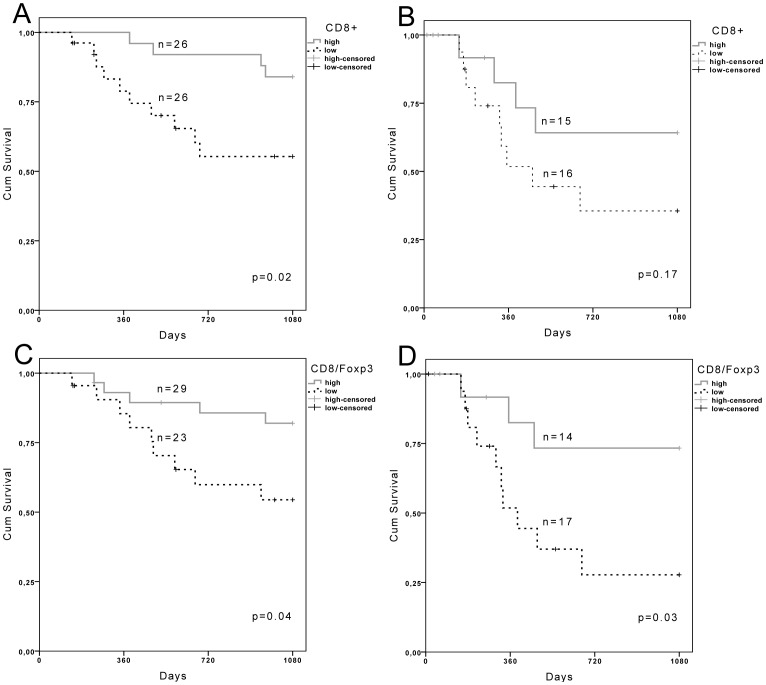
Kaplan-Meier curves showing disease free patient survival depending on TSCC HPV status and TILs, and n denotes the number of patients in each stratified group. A) HPV^+^TSCC and B) HPV^−^TSCC patients stratified by the number of CD8^+^ TILs. High and low denotes CD8^+^ TIL count above and below the median values of 33 for HPV^+^TSCC and 4.4 HPV^−^TSCC. C) HPV^+^TSCC and D) HPV^−^TSCC patients stratified by the ratio of CD8^+^/FoxP3^+^ cell ratio. High and low denotes CD8^+^/FoxP3^+^ cell ratio above and below 1.

### Patients and Material

The cohort consisted of all patients, diagnosed 2000–2006 with TSCC (ICD-10 C09.0-9), that were treated with the intention to cure at Karolinska University Hospital, Stockholm, Sweden, and had available pre-treatment paraffin embedded tumor biopsies. Treatment consisted of accelerated radiotherapy (RT)(1.1+2.0 Gy/day for 4.5 weeks, total dose: 68 Gy) or conventional RT (2.0 Gy/day, for 6.5–7 weeks, total dose: 68 Gy) or, in four cases, induction chemotherapy followed by concomitant RT (CRT). After treatment, patients were followed-up by clinical examination every 3 months during the first 2 years and every 6 months the 3rd year.

**Table 4:** Univariate and multivariate analyses of progonostic factors for 3-year disease free survival in patients with TSCC.

	*Univariate*			*Multivariate*		
	HR	95% CI	p-value	HR	95% CI	p-value
**HPV^+^TSCC**						
*CD8^+^*	0.27	(0.09–0.88)	0.029	0.28	(0.084–0.91)	0.034
*stage*	0.85	(0.11–6.48)	0.88			1
*age*	5.5	(1.55–19.6)	0.008	5.58	(1.5–20.4)	0.009
*sex*	0.83	(0.27–2.62)	0.75	0.68	(0.21–2.22)	0.52
**HPV^-^TSCC**						
*CD8^+^/Foxp3^+^*	0.27	(0.073–0.99)	0.048	0.21	(0.057–0.81)	0.023
*stage*	6.91	(0.89–53.6)	0.064	8.9	(1.12–70.6)	0.039
*age*	0.74	(0.23–2.4)	0.62	0.64	(0.18–2.31)	0.50
*sex*	1.08	(0.56–2.05)	0.83	0.92	(0.46–1.87)	0.83

Abbreviations: HR, hazard ratio; CI, confidence interval.

Data on the presence of high risk HPV types (presence of HPV DNA by PCR) and p16^INK4a^ status (by immunohistochemistry) were obtained from previous studies [3], [7], [22] and patients were divided into 4 groups (A-D) at baseline (for further details see Tables 1 and 2); (A) a random sample (n=31) of all patients (n=109) with HPV^+^, p16^INK4a^ positive (>75% p16^INK4a^ positive tumor cells) (p16^+^) tumors with a good clinical outcome (defined as no relapse and alive 3 years after diagnosis); (B) all patients (n=21) with HPV^+^, p16^+^ tumors with a poor clinical outcome (defined as relapsed in disease and/or dead of disease within 3 years after diagnosis); (C) all patients (n=11) with HPV^−^, p16^INK4a^ negative (p16^−^) tumors with a good clinical outcome; (D) all patients (n=20) with HPV^−^, p16^−^ tumors with a poor clinical outcome (Table 1). A sample of 31 of the 109 original tumors in group A was chosen to obtain a more similar number of patients from each group for further studies of TILs, since this group dominated with 68% of the total number of tumors. Patients with HPV^+^, p16^−^ (n=17) and HPV^−^, p16^+^ tumors (n=4) were excluded from further analysis to obtain homogenous groups and to only include HPV^+^ TSCC expressing HPV E6/E7, since p16^INK4a^ expression in HPV^+^ tumors is considered an indication of E7 expression [23]. Patients dying of other causes than TSCC were also excluded. All HPV^+^ TSCC were HPV16^+^ with exception of one tumor with HPV33 and one with HPV56.

### Immunohistochemistry

A standard streptavidin-biotin peroxidase method was employed on 4 µm formalin fixed, paraffin embedded, sections using the mouse monoclonal antibodies anti-CD8 (dilution, 1∶40; clone 4B11; Novocastra Laboratories) and anti-Foxp3 (dilution 1∶100, clone 236A/E7; eBioscience). All sections were subsequently incubated with biotinylated secondary anti-mouse antibody (1∶200, Vector Laboratories, Burlingame, CA, U.S.A.) followed by incubation with the avidin-biotin-complex-PO using the VECTASTATIN® Elite® ABC kit (Vector Laboratories) and developed in DAB.

The CD8^+^ and Foxp3^+^ tumour lymphocyte infiltration was evaluated by two researchers, blinded for clinical outcome, each counting the cells in 10 randomly selected high-power fields (40×) per sample. The mean value was reported for each tumor. The ratio of tumor infiltrating CD8^+^ and Foxp3^+^ cells was calculated for each individual tumor, the mean of these ratios was then calculated per subgroup.

### Statistical Analysis

Tests for comparisons of continuous data were performed using linear regression models. Results were presented as mean differences or odds-ratios together with 95% confidence interval. The comparisons of patient and tumor characteristics between the different groups in Tables 1 and 2 were performed using Fisher’s exact test (two-tailed). All these analysis were performed in the STATA11 (StataCorp). A p-value≤0.05 was considered as significant.

Disease-free survival (DFS) was defined as time from the date of diagnosis to the date of the last known occasion that the patient was disease-free or the date of disease recurrence (local, regional or distant recurrence). Death without documented recurrence was censored at the date of death. Kaplan-Meier curves were used to present survival data and the log-rank test was used to compare survival curves. In the multivariate analyze, a Cox proportional hazards model was used to adjust for covariates. Two-sided p-values were reported. These analyses were performed in SPSS (IBM SPSS Statistics, v20).

## Results

### Clinical and Pathological Parameters of Patients and Tumors

Details of all patients and their tumors are shown in Tables 1 and 2. As described above, patients were divided into 4 study groups depending on tumor HPV status and clinical outcome within 3 years. The only significant difference between the different study groups was that patients with HPV^−^tumors and good clinical outcome had a lower clinical stage than patients with poor clinical outcome (p=0.005).

There were no differences with regard to any of the studied clinical parameters between the sample of 31 patients and the total population of 109 patients with HPV^+^ TSCC and good clinical outcome (Table 2).

### The Number of CD8^+^ TILs Correlates to HPV Status and Clinical Outcome for Patients with TSCC

TSCC patients with a good clinical outcome had a significantly higher number of CD8^+^ TILs in their tumors (Figure 1), than those with poor clinical outcome, independent of tumor HPV status (mean 50.4 and 12.8 resp., p<0.001), (Table 3 and Figure 2A). Furthermore, when stratifying for HPV status, HPV^+^TSCC, from patients with a good clinical outcome, had more CD8^+^ TILs than those from patients with poor clinical outcome (mean 61.4 and 21.0 resp., p=0.002), (Table 3). The mean number of CD8^+^ TILs was also significantly higher in HPV^−^TSCC from patients with good clinical outcome, when compared to HPV^−^TSCC from patients with poor clinical outcome (mean 19.4 and 4.7 resp., p=0.001).

Moreover, HPV^+^TSCC had a significantly higher mean number of tumor infiltrating CD8^+^ lymphocytes compared to HPV^−^TSCC (mean 45.6 and 9.9 resp., p<0.001) (Figure 2A). This difference was still significant when adjusted for prognosis (p=0.001).

To study the correlation between CD8^+^ TILs and DFS in HPV^+^ TSCC patients, a Kaplan-Meier analysis was performed. HPV^+^ and HPV^-^TSCC patients were dichotomized based on the median value (33 and 4.4 respectively) of CD8^+^ TILs. As demonstrated in Figure 3A patients with HPV^+^ tumors and a high number of CD8^+^ TIL had a cumulative survival of 84% compared to 55% for patients with low number of CD8^+^ (p=0.02). Similar cumulative survival trends (64% and 36%, respecitvely) were also observed for HPV^-^ patients, although these differences were not statistically significant (Figure 3B). Notably, for patients with HPV^+^TSCC the Kaplan-Meier curves were calculated on the sample of TSSC belonging to group A and all TSCC from group B (Figure 3A).

### The Number of Foxp3^+^ TILs Correlates to HPV Status in TSCC

A significantly higher number of Foxp3^+^ TILs (Figure 1) were observed in HPV^+^TSCC as compared to that in HPV^−^TSCC (mean 33.2 and 14.7, resp., p<0.001) (Figure 2B). When adjusting for prognosis, this difference was still significant (p=0.001). However, no differences in the levels of Foxp3^+^ TILs were observed in tumors from patients with a good or poor clinical outcome (mean 27.9 and 24.6, resp.) (Table 3).

### A High CD8^+^/Foxp3^+^ Ratio is Linked to a Good Clinical Outcome in Patients with TSCC

As a measurement of the relative number of cytotoxic and regulatory T-cells in the tumors, the ratio of tumor infiltrating CD8^+^ and Foxp3^+^ cells was calculated for each tumor. A high CD8^+^/Foxp3^+^ cell ratio is an indication that the regulatory T-cells are in a minority and thus less likely to overshadow the function of cytotoxic T-cells. For patients with TSCC a high CD8^+^/Foxp3^+^ ratio was positively correlated to a good clinical outcome when adjusted for prognosis (p=0.04) (Table 3). However, the difference related to prognosis in CD8^+^/Foxp3^+^ TIL ratio was not significant when patients with HPV^+^TSCC or HPV^−^TSCC were analyzed separately, although the tendency was similar to that observed for TSCC patients in general.

To study the correlation between a high/low CD8^+^/Foxp3^+^ TIL ratio and DFS for TSCC, a Kaplan-Meier analysis was performed. HPV^−^ and HPV**^+^**TSCC patients were dichotomized based on a CD8^+^/Foxp3^+^ TIL ratio above or below 1. As demonstrated in Figure 3D patients with HPV^−^TSCC and a high CD8^+^/Foxp3^+^ TIL ratio had a cumulative survival of 73% as compared to 28% for patients with a low CD8^+^/Foxp3^+^ TIL ratio (p=0.03). Similarly, HPV**^+^**TSCC patients with a high CD8^+^/Foxp3^+^ TIL ratio had a higher cumulative survival (82%) than those with a low CD8^+^/Foxp3^+^ TIL ratio (54%, p=0.04) (Figure 3C).

### Correlation between Clinical Parameters, CD8^+^ and Foxp3^+^ Cells and 3-year DFS in Patients with TSCC

When CD8^+^ and Foxp3^+^ TILs values for all TSCC, stratified for HPV status, were analyzed for age, sex or clinical stage, no correlation was found (data not shown).

Hazard ratios (HR) were calculated separately for HPV^+^ and HPV^−^TSCC, using both univariate and multivariate tests (Table 4). For HPV^+^TSCC the hazard ratio for a high number of CD8^+^ cells was 0.27 (p=0.029), while for HPV^−^TSCC the hazard ratio for a high CD8^+^/Foxp3^+^ ratio was 0.27 (p=0.048). To determine whether CD8^+^ cells and CD8^+^/Foxp3^+^ ratio were independent prognostic factors, a multivariate Cox analysis was performed with clinical stage, age and sex as covariates (Table 4). For HPV^+^TSCC, both the number of CD8^+^ cells and age were independently significantly correlated to 3-year DFS (p=0.034 and p=0.009, resp.). For HPV^−^TSCC both CD8^+^/Foxp3^+^ ratio and stage was significantly independently correlated to 3-year DFS (p=0.023 and p=0.039, resp.). Similarly, a high CD8^+^/Foxp3^+^ ratio correlated to a 3-year DFS both in the univariate and multivariate analyzes also for HPV^+^TSCC (HR 0.33, p=0.048 and HR 0.23, p=0.015 respectively) (data not shown).

## Discussion

In this study, we found a significant correlation between a high CD8^+^ T-cell infiltration and clinical outcome for both patients with HPV^+^ and HPV^−^TSCC, as well as between a high CD8^+^/Foxp3^+^ TIL ratio and disease free survival for both patients with HPV^+^ and HPV^−^TSCC. In addition, we demonstrated a correlation both between tumor infiltrating CD8^+^ and Foxp3^+^ T-cells to HPV status in TSCC.

The fact that CD8^+^ infiltration was more pronounced and influenced prognosis positively in this study for HPV^+^TSCC, may be an important reason for why the majority of HPV^+^TSCC have a good clinical outcome in comparison to HPV^−^TSCC [6], [7]. This would be in line with experimental studies showing the importance of the immune system for combating HPV^+^ head and neck squamous cell carcinoma (HNSCC) [24]. The result is also in concordance with many studies on tumors from other sites e.g. for colon and cervical cancer [13], [14], [15], [18]. Notably, in cervical cancer, where the vast majority of tumors are HPV^+^, both a high number of CD8^+^ TIL and a high CD8^+^/regulatory T-cell ratio were correlated to the absence of lymph node metastasis [14]. A similar result has also been published for HNSCC, where Ogino and colleagues demonstrate a positive correlation between a high CD8^+^ T-cell infiltration and cause specific survival [25]. In addition, the presence of HPV16 specific T-cells in HPV^+^ HNSCC was recently demonstrated, although correlation to prognosis was not investigated [26]. The strong positive correlation between CD8^+^ TILs and good clinical outcome for patients with TSCC indicates that assessment of CD8^+^ TILs could be used as an additional prognostic biomarker in combination with tumor HPV status.

We also demonstrated that a high CD8^+^/Foxp3^+^ T-cell ratio significantly correlated to a disease free survival in both patients with HPV^+^ and HPV^−^TSCC. Previous publications have linked Foxp3^+^ TILs to a worse prognosis in e.g. ovarian and breast cancer [16], [18]. Furthermore, a high percentage of Tregs has been correlated to a poor 5-year survival in cervical cancer [27]. However, there are also instances where Foxp3^+^ TILs have been linked to a favorable prognosis such as e.g. in colon cancer, Hodgkin lymphoma and HNSCC [20], [21], [28]. The result obtained in the present study indicates that infiltrating T-cells also are of importance for clinical outcome for patients with HPV^−^TSCC, although possibly to a lesser extent. The assessment of CD8^+^/Foxp3^+^ T-cell ratio could thus also be used as a clinical prognostic marker for patients with HPV^+^ or HPV^−^TSCC.

Importantly, estimating CD8^+^ and Foxp3^+^ TILs by IHC can be performed at pathology units in hospitals. The HPV status of TSCC is already assessed routinely in some clinics, e.g. at the Karolinska University Hospital in Stockholm, and if used in conjunction with evaluation of CD8^+^ and Foxp3^+^ TILs in pathology units, a better prediction of clinical outcome may be obtained. However, the size of the field evaluated and cut-off values have to be precisely defined.

In addition, we observed that HPV^+^TSCC had a significantly higher infiltration of both CD8^+^ and Foxp3^+^ cells than HPV^−^TSCC. This was not unexpected given that HPV^+^TSCC expresses foreign viral antigens. In fact, the data indicate that, although the immune defense has failed to impede the development of the HPV^+^TSCC, there is still an immunological difference between HPV^+^ and HPV^−^TSCC.

It is important to note that the presence of TILs in TSCC is not enough for tumor rejection. However, it has been demonstrated in a mouse model that an intact immune defense is important for a complete tumor clearance upon radiation treatment [24]. Furthermore, it is possible that irradiation may activate the immune response both against remaining viable cells of the original tumor and against lymph node metastasis.

Recently, many tumor biomarkers have been explored with regard to prognosis for patients with HNSCC [29], [30], [31], [32]. However, in most of these studies the HPV status of the tumors was not taken into account and often tumors from different HNSCC subsites were grouped together when biomarkers were correlated to prognosis. The results of the present study demonstrate the importance of separating tumors depending on HPV status. In addition, in studies of this kind, the tumors should also be stratified depending on tumor subsite, since e.g. the overall survival for patients with HNSCC differs depending on both HPV status and tumor subsite.

There are some limitations in the present study. Firstly, it is a retrospective study with all patients diagnosed with TSCC in Stockholm during 2000–2006 and treated with intention to cure. However, it is important to note that treatment during this period was standardized with conventional/accelerated RT. In addition, previous publications, analyzing the clinical outcome of the patients included in the present study, have failed to demonstrate differences in clinical outcome for patients with oropharyngeal cancers depending on treatment [33], [34].

Secondly, most TSCC today are HPV^+^ with a favorable clinical outcome for the patients. Hence, this group was reduced in the present study by randomization. On the other hand, we were not able to demonstrate any significant differences between the study sample and the total material in this group. Another limitation is the few numbers of patients in the HPV^−^ groups, although all patients were included with this feature. To better study the HPV^−^TSCC population, other medical centers should be included.

Finally, we have not assessed if the tumor infiltrating CD8^+^ and Foxp3^+^ cells are targeted against specific tumor associated antigens or assayed for functional CD8^+^ using e.g. granzyme B. Furthermore, since Foxp3, although at low levels, may be transiently induced in CD4^+^ and CD8^+^ T-cells upon stimulation, all Foxp3^+^ TILs may not be Tregs [35]. However, for the purpose of this study, i.e. to find clinically relevant and easily identifiable markers, this was not of importance, although it would be of interest to address these questions in other studies.

In conclusion, tumor infiltrating CD8^+^ and Foxp3^+^ cells in TSCC display different profiles depending on tumor HPV status and clinical outcome. Thus, combining the presence of CD8^+^ T-cells and/or CD8^+^/Foxp3^+^ cell ratio with HPV status, may be of additive prognostic value and for better individualizing patient treatment.
